# Analysis of *Marchantia polymorpha*–microorganism interactions: basis for understanding plant–microbe and plant–pathogen interactions

**DOI:** 10.3389/fpls.2024.1301816

**Published:** 2024-02-07

**Authors:** Jorge Poveda

**Affiliations:** Recognised Research Group AGROBIOTECH, UIC-370 (JCyL), Department of Plant Production and Forest Resources, Higher Technical School of Agricultural Engineering of Palencia, University Institute for Research in Sustainable Forest Management (iuFOR), University of Valladolid, Palencia, Spain

**Keywords:** bryophytes, plant immunity, marchantin A, Pseudomonas syringae, Fusarium oxysporum, Trichoderma

## Abstract

*Marchantia polymorpha* is a bryophyte gaining significance as a model plant in evolutionary studies in recent years. This is attributed to its small-sequenced genome, standardized transformation methodology, global distribution, and easy and rapid *in vitro* culturing. As an evolutionary model, *M. polymorpha* contributes to our understanding of the evolution of plant defensive responses and the associated hormonal signaling pathways. Through its interaction with microorganisms, *M. polymorpha* serves as a valuable source of knowledge, yielding insights into new microbial species and bioactive compounds. Bibliographic analysis involved collecting, reading, and categorizing documents obtained from the Scopus and Web of Science databases using different search terms. The review was based on 30 articles published between 1995 and 2023, with Japanese and Spanish authors emerging as the most prolific contributors in this field. These articles have been grouped into four main themes: antimicrobial metabolites produced by *M. polymorpha*; identification and characterization of epiphytic, endophytic, and pathogenic microorganisms; molecular studies of the direct interaction between *M. polymorpha* and microorganisms; and plant transformation using bacterial vectors. This review highlights the key findings from these articles and identifies potential future research directions.

## 1 Bryophytes as a source of microorganisms

Bryophytes (Bryophyta division) constitute a highly diverse group of terrestrial plants, comprising over 23,0000 species distributed worldwide. Taxonomically, this plant group is classified into three distinct categories: liverworts (class Hepaticopsida), hornworts (class Anthocerotopsida) and mosses (class Bryopsida). Bryophytes have significant ecological importance globally, thriving in a wide variety of habitats (Bahuguna et al., 2013). This plant group has noteworthy applications, including the extraction of bioactive compounds for the pharmaceutical industry, the production of substrates and materials for horticultural cultivation, the generation of solid fuels, architectural and decorative purposes, and, notably, serving as an indicator of environmental conditions (Saxena & Harinder, 2004).

Liverworts, hornworts, and mosses collectively represent a group of model plants in the study of how plants successfully colonized terrestrial environments and evolved into vascular plants. The transition from aquatic to terrestrial habitats, known for their aggressiveness, requires the development of various evolutionary strategies by ancestors of modern bryophytes. To safeguard themselves from intense UV radiation, bryophytes present protective metabolites, whereas against desiccation, they form a hydrophobic cuticle or a minimal cuticle with the prevalence of phenolic compounds and waxy components. Additionally, the establishment of ancestors of modern bryophytes in this new and harsh terrestrial environment may have involved the development of beneficial interactions with microorganisms along with the synthesis of biocidal compounds to ward off potential pathogens and herbivores. However, this aspect is not yet fully understood and similar biotic stresses may exist in aquatic life (Degola et al., 2022).

Approximately 450 million years ago, plants successfully colonized terrestrial environments, and contemporary studies increasingly highlight the pivotal role of plant-microorganism interactions in this process. It has been suggested that fungi, specifically arbuscular mycorrhizal fungi within the Glomeromycotina and Mucoromycotina subphyla, constitute the fundamental group of microorganisms involved. Their symbiotic relationship with plants necessitates the development of specialized fungal structures for nutritional exchange, known as arbuscules (Reboledo and de León, 2021).

The diversity and biological activity of endophytic microorganisms isolated from bryophytes represent topics of significant scientific interest. In 2023, the term “bryendophytes” was coined to collectively refer to these microorganisms, mainly bacteria and fungi (Stelmasiewicz et al., 2023a). Bacteria play a key role in the presence and diversity of endophytes across various habitats, because of the different biological activities they engage in during their interactions with bryophytes. The main functions of bryophyte endophytic bacteria include atmospheric nitrogen fixation, production of antifungal and antibacterial compounds against plant pathogens, and decomposition of colonized tissues once the host plant dies (Koua et al., 2015; Glime, 2022). Recently, additional functions have been described, such as the fixation of atmospheric carbon dioxide by cyanobacteria associated with bryophytes (Jassey et al., 2022).

Both bacteria and bryophyte–endophyte fungi form crucial symbiotic relationships for the possible establishment of these plants in extreme habitats. For instance, the utilization of bryophytes in the restoration of desertified areas requires a prolonged tolerance. Specific bacterial and fungal communities have been described in bryophyte species that can thrive in challenging environments (Cao et al., 2020). In addition, it has been identified that the main bacterial and fungal mechanisms employed to enhance bryophyte tolerance to drought involve an increase in proline content, as well as superoxide dismutase and peroxidase activity in plant tissue (Cao et al., 2020). Just as bryophytes form associations with specific endophytic microorganisms under dry conditions, they also form associations under environmental contamination. Consequently, the study of bryophyte diversity and its associations with protists and bacteria provides insights into the level of atmospheric contamination in each location (Meyer et al., 2010; Meyer et al., 2012).

Finally, an aspect of considerable interest, yet currently underexplored, is the utilization of bryophytes as a source of microorganisms to improve crops and/or forest species. Bryophytes are known to thrive in soils deficient in specific nutrients owing to the microorganisms associated with them, such as silicon-poor soils. In a study conducted with the bryophyte *Hypnum plumaeforme*, a silicate-solubilizing bacterium identified within the *Kosakonia* genus was isolated from rhizoids (Hu et al., 2019). Subsequently, the root inoculation of maize seedlings with this bacterium resulted in increased plant growth and tissue accumulation of silicon. This enhancement was attributed to the release of silicon from feldspar and quartz powder facilitated by the bacteria (Hu et al., 2019).

## 2 *Marchantia polymorpha*: a bryophyte model

Historically, the liverwort species *Marchantia polymorpha* has often been used as a model plant (Bowman, 2016; Bowman et al., 2016). In the 19th century, the anatomy of various tissues and organs in *M. polymorpha* gametophores and sporophytes was described and illustrated with remarkable quality, albeit with some limitations owing to the techniques and knowledge available at that time (Leitgeb and Schuster, 1879; Mirbel, 1835). In the late 20th century, *M. polymorpha* became the first plant species for which the organellar genomes of chloroplasts and mitochondria were sequenced (Ohyama et al., 1986; Oda et al., 1992). However, it was later shown that the initially sequenced genome belonged to a different *Marchantia* species mixed in the culture used (Bowman, 2022). Subsequently, the sex chromosome (male: V, formerly called Y) was fully sequenced for the first time in plants (Yamato et al., 2007). More recently, sequencing of the remaining nuclear genome has highlighted the important evolutionary position and advantages for genetic studies of *M. polymorpha*, reinforcing its status as a model plant (Bowman et al., 2017; Montgomery et al., 2020; Iwasaki et al., 2021).

Embryophytes (land plants) are believed to have colonized terrestrial environments approximately 470 million years ago, diverging from a common ancestor into tracheophytes (vascular plants) and bryophytes (non-vascular plants) (Morris et al., 2018). Bryophytes are phylogenetically classified into three major groups: hornworts, mosses, and liverworts. Recent large-scale transcriptomic analyses across species, exemplified by the 1KP project (One Thousand Plant Transcriptomes Initiative, 2019), have supported the assumption that bryophytes are monophyletic (Wickett et al., 2014; de Sousa et al., 2019; Harris et al., 2020). Within bryophytes, hornworts are sister to a monophyletic clade of mosses and liverworts, known as setaphytes (Puttick et al., 2018). Despite the generally small genome size of bryophytes, there is evidence of whole-genome duplications in the genome of the moss *Physcomitrium patens*, but not in the hornwort *Anthoceros agrestis* or the liverwort *M. polymorpha* (Bowman et al., 2017; Lang et al., 2018; Li et al., 2020). Furthermore, despite low genetic redundancy, the *M. polymorpha* genome shares many genes regulating growth, development, stress responses, and other functions with other land plants. This enables the study of the common mechanisms in simplified models (Bowman et al., 2017). In addition, bryophytes, such as *M. polymorpha*, represent good model plants for the study of horizontal gene transfer during the evolution of plant species. This assertion is based on the premise that there is significant horizontal gene transfer between the ancestral charophyte (common ancestor of land plants) and ancestral bryophyte (common ancestor of bryophytes), which explains the evolutionary success of land plant colonization (Ma et al., 2022).

The fully assembled genome of *M. polymorpha*, consisting of eight autosomes and a sex chromosome (U/V), has been sequenced for Takaragaike-1 (Tak-1, male) and Takaragaike-2 (Tak-2, female), and the sequences are available at MarpolBase (https://marchantia.info/). In addition to genomic information, well-established *Agrobacterium*-mediated genetic transformation techniques are also available (Ishizaki et al., 2008; Kubota et al., 2013; Tsuboyama and Kodama, 2014; Tsuboyama et al., 2018). Vector toolkits offer various options including promoters, intracellular targeting signals, tags (such as fluorescent proteins), and selection markers (Ishizaki et al., 2016; Sauret-Güeto et al., 2020; Westermann et al., 2020). Genetic analysis can be facilitated through targeted genome modification using homologous recombination and CRISPR-Cas9 genome-editing techniques (Ishizaki et al., 2013; Sugano et al., 2014). Owing to these capabilities and ease of handling in laboratories, *M. polymorpha* is valuable for investigating fundamental plant biology in many aspects, despite its morphological features differing from those of angiosperms. These features include a creeping leafy thallus structure with dorsal air chambers but lack stomata (Bowman, 2022).

Therefore, *M. polymorpha* represents a model plant of great interest in the study of the evolutionary mechanisms governing the physiology of present-day land plants. In addition, microorganisms play a fundamental role in plant evolution from terrestrial to aquatic life, and bryophytes are a fundamental focus of study to understand how beneficial and pathogenic plant–microorganism interactions occur. Accordingly, considering the important evolutionary role of bryophyte–microorganism interactions and the establishment of *M. polymorpha* as a model plant, this review focuses on studies examining *Marchantia*’s interactions with microbes and pathogens.

## 3 *M. polymorpha* in plant immunity studies

Reports have indicated that *M. polymorpha* is susceptible to various pathogens, including bacteria, oomycetes, and fungi. Further details on these infections are provided in *Section 5*. Here, we delve into the innate immunity mechanisms of *M. polymorpha* and draw comparisons with those observed in other plant species.

Plant innate immunity is triggered by direct interactions with pathogens in the plasma membrane. Pattern recognition receptors (PRRs) located on the membrane sense a broad spectrum of pathogen-associated molecular patterns (PAMPs), such as bacterial flagellin, Elongation Factor-Tu (EF-Tu), oomycete glucans, and fungal chitin, which activate downstream signaling cascades (Zhang and Zhou, 2010). In the angiosperm *Arabidopsis*, for example, the perception of flagellin by the PRR AtFLS2 induces the production of reactive oxygen species (ROS), mitogen-activated protein kinases (MAPKs), growth inhibition, and expression of pathogen defense genes (Gómez-Gómez et al., 1999). Similarly, the PRRs AtEFR and AtCERK1 are known to perceive bacterial EF-Tu and fungal chitin, respectively (Zipfel et al., 2006; Miya et al., 2007; Wan et al., 2008). However, the genome of *M. polymorpha* does not encode homologs of AtFLS2 or AtEFR, but does encode homologs of AtCERK1 and other related lysine motif (LysM) domain-containing receptors (Bowman et al., 2017; Yotsui et al., 2023). Indeed, treatment with chitin induced ROS production in *M. polymorpha*, whereas synthetic peptides derived from flagellin and EF-Tu (flg22 and elf18, respectively) did not elicit any defense responses (Gimenez-Ibanez et al., 2019; Chu et al., 2023; Yotsui et al., 2023). Nevertheless, treatment with the crude extract of the bacterial pathogen *Pseudomonas syringae* pv. tomato (*Pto*) DC3000 caused growth inhibition and expression of PAMP-responsive genes in *M. polymorpha* (Gimenez-Ibanez et al., 2019), suggesting that PRRs are distinct from AtFLS2 and AtEFR homologs. Interestingly, loss of function of the AtCERK1 homolog MpLYK1 showed hypersusceptibility to *Pto* DC3000 (Yotsui et al., 2023). Further investigation is required to elucidate PRRs and ligand PAMPs in *M. polymorpha*.

Pathogens adapted to overcome these PAMP-triggered immunities (PTIs) by delivering various effector proteins into the host cells. Plants have evolved nucleotide-binding site leucine-rich repeat (NBS-LRR) type receptors for effector proteins as the second layer of pathogen recognition in the cytosol, activating effector-triggered immunity (ETI) (Zhang and Zhou, 2010). In *M. polymorpha*, it has been demonstrated that various pathogens, including bacterial Pto DC3000, oomycete *Phytophthora palmivora*, and fungal *Fusarium oxysporum*, secrete effector proteins to suppress PTIs and promote disease (Carella et al., 2018; Gimenez-Ibanez et al., 2019; Redkar et al., 2022a; Redkar et al., 2022b). Although it is still unclear whether *M. polymorpha* recognizes these effectors, the genome encodes candidates for *NBS-LRR* genes (Xue et al., 2012; Shao et al., 2019) that might be involved in ETIs.

PPRs transmit signals that recognize pathogens to intracellular downstream components through PRR-associated proteins and receptor-like cytoplasmic kinases (RLCKs). In *Arabidopsis*, one extensively studied RLCK is botrytis-induced kinase 1 (AtBIK1), which phosphorylates the NADPH oxidase AtRBOHD, initiating ROS production and phosphorylating MAPK cascades to induce the expression of defense-related genes. Recent studies have revealed that a BIK1 homolog in *M. polymorpha*, MpPBLa (AvrPphB susceptible 1-like a), phosphorylates an RBOHD homolog and MAPKs during chitin-induced immunity (Chu et al., 2023; Yotsui et al., 2023). Whether the PBL family plays similar roles in other PAMP-triggered immune responses in *M. polymorpha* remains unclear.

Angiosperm studies have demonstrated that *M. polymorpha* infected with bacterial, fungal, and oomycete pathogens express several orthologous genes that serve as defense markers (Gimenez-Ibanez et al., 2019; Carella et al., 2019; Redkar et al., 2022; Yotsui et al., 2023). Loss of function of the MpLYK1 receptor abolished the expression of defense-related genes induced by chitin treatment (Yotsui et al., 2023), suggesting that such transcriptional regulation is PTI-dependent. Transcriptomic analyses have also shown that *M. polymorpha* and angiosperm species share several common pathogen-responsive genes (Carella et al., 2019). For example, the transcription factor, MpMyb14, regulates the genes involved in the biosynthesis of phenylpropanoids (flavonoids) required for defense against the oomycete *P. palmivora* (Carella et al., 2019). Importantly, the signaling of the phytohormone salicylic acid (SA) and jasmonate (JA) signaling regulate the expression of pathogen-responsive genes in *M. polymorpha*, similar to angiosperms. Notably, *M. polymorpha* uses dinor-12-oxo-10,15(Z)-phytodienoic acid (dn-OPDA), a precursor of JA in angiosperms (Monte et al., 2018). Bacterial infection by *Pto* DC3000 induced SA accumulation and SA marker gene expression, which were suppressed by treatment with dn-OPDA (Gimenez-Ibanez et al., 2019). In contrast, dn-OPDA is required to defend against the fungal pathogen *Irpex lacteus*, promoted by SA treatment (Matsui et al., 2020). These findings suggest that antagonistic interactions between SA and dn-OPDA(JA) signaling pathways emerged in the common ancestor of bryophytes and vascular plants. It is hoped that further studies on how *M. polymorpha* defends itself against each pathogen will provide insights into the conserved mechanisms of immunity.

## 4 Analysis conducted

A literature review was conducted, along with a quantitative analysis of publications based on the year, journal and country. The compilation of all publications was carried out using the following keywords “*Marchantia polymorpha* AND microorganism,” “*Marchantia polymorpha* AND virus,” “*Marchantia polymorpha* AND bacteria,” “*Marchantia polymorpha* AND fungus,” “*Marchantia polymorpha* AND protist,” and “*Marchantia polymorpha* AND nematode.” The Web of Science™ (Web of Science Core Collection—WoS) (https://www.webofscience.com) and Elsevier^®^ Scopus library services metabase (www.scopus.com) were utilized. This choice was made because of the advantages of scientific rigor compared to other free and open databases, such as Google Scholar (Martín-Martín et al., 2021).

In WoS, after searching the keywords in “All Fields,” without time restrictions, 82 results were retrieved (search conducted on 7 July 2023). Of these 82 articles, 60 were not related to the subject, and two other articles were reviews; therefore, 20 articles were included in the review. This review focuses on the use of *M. polymorpha* as a model plant in evolutionary studies with microorganisms (Poveda, 2020a) and on the identification and metabolic characterization of endophytic microorganisms associated with bryophytes or “bryendophytes” (Stelmasiewicz et al., 2023a). On the other hand, after searching for keywords in “Title, Abstract and Keywords,” without time restrictions, a total of 95 results were retrieved in Scopus (search conducted on 7 July 2023). Of these, 64 were not related to the subject of this work, and one other article was a review (Poveda, 2020a), therefore, 30 articles were included in the review. It is important to note the overlapping results of the two databases. Of the 20 articles from WoS and 30 from Scopus, 20 coincided, with Scopus contributing 10 unique articles. Therefore, the total number of final articles for the review of the *M. polymorpha*–microorganism interaction study was 30.

**Figure 1:**
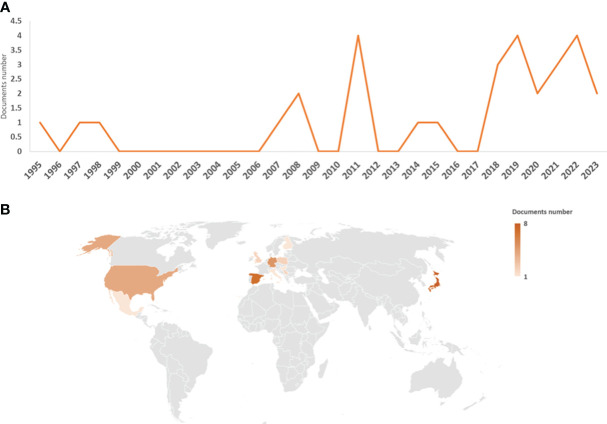
Graphical representation of the data obtained from the bibliographic search. Number of articles per year **(A)** and countries of authors **(B)**; the legend represents the number of articles per country.

The first work on *M. polymorpha*–microorganism interactions was published in 1995 (Kámory et al., 1995), with two additional works published in 1997 (Nasu et al., 1997) and 1998 (Frame et al., 1998). However, there was a gap in the number of publications until 2007 (Kutschera et al., 2007). From 2007 to 2015, several works were published (totaling nine articles), with the highest number of publications being 2008 (two articles) and 2011 (four articles). Since 2018, there has been a clear increase and stabilization in the number of publications, with at least two to four articles per year (**Figure 1A**). With respect to the country of article publication, Japan stood out as the country with the most articles with authors (eight articles). Next in importance are Spain, Germany, and the USA had seven, five, and four articles, respectively. The fifth, sixth, and seventh places are Poland, Serbia, and the UK (with two articles). With one publication with authors from this position we found a total of six countries from Europe (Finland, Hungary, and Italy), America (Mexico and Puerto Rico), Africa (South Africa), and Asia (Turkey) (**Figure 1B**).

The authors have published their works in 23 different journals. Notably, *Plant* & *Cell Physiology* (Oxford Academic) stands out with four articles, *Archives of Phytopathology and Plant Protection* (Taylor & Francis), *Molecules* (MDPI), and *Symbiosis* (Springer), each featuring two articles. The remaining journals only published one article (**Table 1**). Regarding the number of citations per article, the most referenced article was by Ishizaki et al. (2008), accumulating 220 citations in Scopus. However, these articles did not have a high number of citations compared to other articles related to plant–microbe interactions. For instance, the second most-cited article, over 25 years old, has garnered only 45 citations in Scopus. Among the top 10 most-cited articles, two were relatively recent. One, published in *Current Biology* (Gimenez-Ibanez et al., 2019), received 26 citations in WoS and 25 citations in Scopus. The other, published on *Plant and Cell Physiology* (Matsui et al., 2020), accumulated 25 citations in both WoS and Scopus (**Table 2**).

**Table 1:** Journals where the reviewed papers were published.

Journal	Number of Papers	Paper References
*Plant Cell Physiol.*	4	Ishizaki et al. (2008); Sugano et al. (2014); Matsui et al. (2020); Iwakawa et al. (2021)
*Arch. Phytopathol. Plant Prot.*	2	Gahtori and Chaturvedi (2011); Mewari and Kumar (2011)
*Molecules*	2	Stelmasiewicz et al. (2022); Stelmasiewicz et al. (2023b)
*New Phytol.*	2	Nelson et al. (2018); Redkar et al. (2022b)
*Symbiosis*	2	Nelson and Shaw (2019); Poveda (2020b)
*Curr. Biol.*	1	Gimenez-Ibanez et al. (2019)
*Front. Microbiol.*	1	Tamura et al. (2019)
*Indian J. Tradit. Knowl.*	1	Yayintas et al. (2019)
*Int. J. Syst. Evol. Microbiol.*	1	Schauer et al. (2011)
*J. Biosci. Bioeng.*	1	Nasu et al. (1997)
*J. Med. Plant Res.*	1	Sabovljević et al. (2011)
*J. Serb. Chem. Soc.*	1	Ivković et al. (2021)
*Naturwissenschaften*	1	Kutschera et al. (2007)
*P. R. Health Sci. J.*	1	Frame et al. (1998)
*Pharmaceuticals*	1	Jimenez-Aleman et al. (2021)
*Pharm. Biol.*	1	Mewari and Kumar (2008)
*Plan Cell*	1	Redkar et al. (2022a)
*Plant Commun.*	1	Matsumoto et al. (2022)
*Plant Med.*	1	Kámory et al. (1995)
*Planta*	1	Poveda et al. (2023)
*Plos One*	1	Ishizaki et al. (2015)
*Proc. Natl. Acad. Sci.*	1	Carella et al. (2018)
*Sci. Rep.*	1	Alcaraz et al. (2018)

**Table 2:** Number of citations of the 10 most cited articles.

Ranking	Paper reference	Journal	WoS Citations	Scopus Citations
1	Ishizaki et al. (2008)	*Plant and Cell Physiology*	Not indexed	220
2	Frame et al. (1998)	*Puerto Rico Health Sciences Journal*,	Not indexed	45
3	Kámory et al. (1995)	*Planta Medica*	Not indexed	37
4	Carella et al. (2018)	*Proceedings of the National Academy of Sciences of the United States of America*	Not indexed	36
5	Alcaraz et al. (2018)	*Scientific Reports*	32	35
6	Nelson et al. (2018)	*New Phytologist*	33	33
7	Gimenez-Ibanez et al. (2019)	*Current Biology*	26	25
8	Matsui et al. (2020)	*Plant and Cell Physiology*	25	25
9	Schauer et al. (2011)	*International Journal of Systematic and Evolutionary Microbiology*	Not indexed	25
10	Mewari and Kumar (2008)	*Pharmaceutical Biology*	22	25

## 5 *M. polymorpha*–microorganism interactions

**Figure 2:**
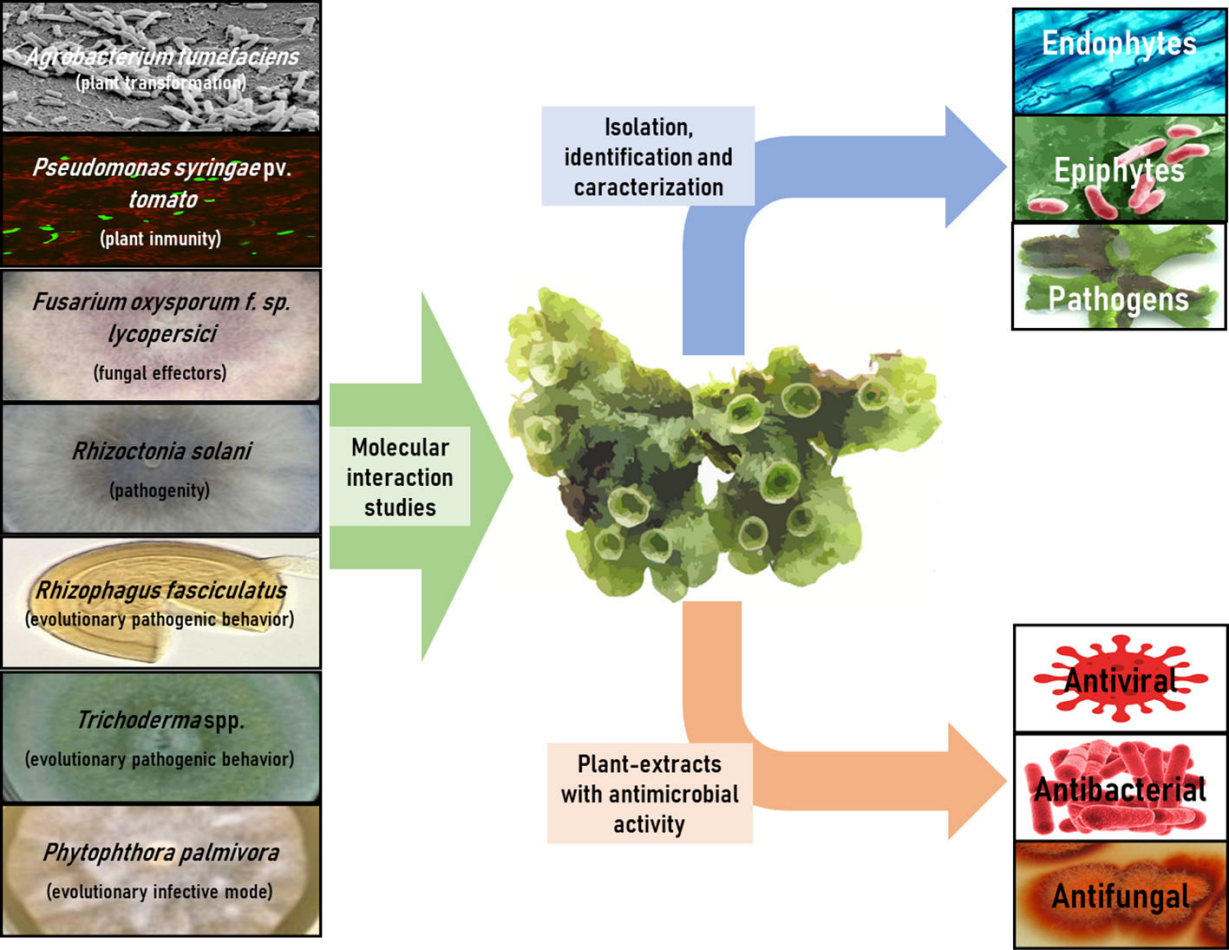
Infographic summary of different types of microbial interactions described for *M. polymorpha*.

The 30 articles analyzed in this review have been meticulously compiled and classified in **Table 3**, based on interacting microorganisms, types of interaction, and key findings. **Figure 2** provides a summary infographic of the different types of microbial interactions described for *M. polymorpha*. The different studies analyzed will be discussed in the following thematic order: antimicrobial metabolites produced by *M. polymorpha*, identification and characterization of epiphytic, endophytic, and pathogenic microorganisms, molecular studies of the direct interaction between *M. polymorpha* and microorganisms, and plant transformation by means of bacterial vectors.

**Table 3:** Compilation of all existing works on *M. polymorpha*–microorganism interaction, indicating the microorganism, the type of interaction, and the main finding.

Interaction Microorganism		Type of Interaction	Main Finding	Reference
Group	Specie			
Virus	Severe acute respiratory syndrome coronavirus 2 (SARSCoV-2)	Indirect: *M. polymorpha* metabolites against virus *in vitro*	*M. polymorpha* metabolites (such as pheophorbide A) have antiviral capacity	Jimenez-Aleman et al. (2021)
Bacteria	*Agrobacterium tumefaciens*	Direct contact	*M. polymorpha* can be stably transformed using *A. tumefaciens* as a vector.	Nasu et al. (1997)
		Direct contact	*M. polymorpha* can be stably transformed using *A. tumefaciens* as a vector.	Ishizaki et al. (2008)
		Direct contact	*M. polymorpha* can be stably transformed using *A. tumefaciens* as a vector.	Sugano et al. (2014)
		Direct contact	*M. polymorpha* can be stably transformed using *A. tumefaciens* as a vector.	Ishizaki et al. (2015)
		Direct contact	*M. polymorpha* can be transient transformed using *A. tumefaciens* as a vector.	Iwakawa et al. (2021)
	*Bacillus subtilis*	Indirect: *M. polymorpha* metabolites against bacteria *in vitro*	*M. polymorpha* methanolic extracts have antibacterial capacity	Yayintas et al. (2019)
		Indirect: *M. polymorpha* metabolites against bacteria *in vitro*	*M. polymorpha* metabolites (such as marchantin A) have antibacterial capacity against Gram-positive bacteria.	Ivković et al. (2021)
	*Clavibacter michiganensis*	Indirect: *M. polymorpha* metabolites against bacteria *in vitro*	*M. polymorpha* metabolites (such as marchantin A) have antibacterial capacity against Gram-positive bacteria.	Ivković et al. (2021)
	*Escherichia coli*	Indirect: *M. polymorpha* metabolites against bacteria *in vitro*	*M. polymorpha* methanol and flavonoid extracts have antibacterial capacity	Mewari and Kumar (2008)
	*Haemophylus influenzae*	Indirect: *M. polymorpha* metabolites against bacteria *in vitro*	*M. polymorpha* metabolites (such as marchantin A) have antibacterial capacity	Kámory et al. (1995)
	*Listeria monocytogenes*	Indirect: *M. polymorpha* metabolites against bacteria *in vitro*	*M. polymorpha* metabolites (such as marchantin A) have antibacterial capacity against Gram-positive bacteria.	Ivković et al. (2021)
	*Methylobacterium* sp.	Direct contact	Discovery of a new epiphytic bacterial species and characterization of cluster formation and promotion of plant growth capacity	Kutschera et al. (2007)
	*M. marchantiae*	Direct contact	Discovery of a new epiphytic bacterial species	Schauer et al. (2011)
	*Mycobacterium smegmatis*	Indirect: *M. polymorpha* metabolites against bacteria *in vitro*	*M. polymorpha* ethanol extracts have antibacterial capacity	Frame et al. (1998)
	*Neisseria meningitidis*	Indirect: *M. polymorpha* metabolites against bacteria *in vitro*	*M. polymorpha* metabolites (such as marchantin A) have antibacterial capacity	Kámory et al. (1995)
	*Pasteurella multocida*	Indirect: *M. polymorpha* metabolites against bacteria *in vitro*	*M. polymorpha* metabolites (such as marchantin A) have antibacterial capacity	Kámory et al. (1995)
		Indirect: *M. polymorpha* metabolites against bacteria *in vitro*	*M. polymorpha* methanol and chloroform extracts have antibacterial capacity	Gahtori and Chaturvedi (2011)
	*Proteus mirabilis*	Indirect: *M. polymorpha* metabolites against bacteria *in vitro*	*M. polymorpha* methanol extracts have antibacterial capacity	Mewari and Kumar (2008)
	*Pseudomonas aeruginosa*	Indirect: *M. polymorpha* metabolites against bacteria *in vitro*	*M. polymorpha* metabolites (such as marchantin A) have antibacterial capacity	Kámory et al. (1995)
	*Pseudomonas syringae* pv. *tomato*	Direct contact	Evolutionary molecular plant–microbe interactions (EvoMPMI) study: land plants share a basic plant immune system against plant–pathogen bacteria	Gimenez-Ibanez et al. (2019)
		Direct contact	To achieve a system for quantifying bacterial tissue colonization in *M. polymorpha* by bioluminescence	Matsumoto et al. (2022)
	*Staphylococcus aureus*	Indirect: *M. polymorpha* metabolites against bacteria *in vitro*	*M. polymorpha* metabolites (such as marchantin A) have antibacterial capacity	Kámory et al. (1995)
		Indirect: *M. polymorpha* metabolites against bacteria *in vitro*	*M. polymorpha* methanol and flavonoid extracts have antibacterial capacity	Mewari and Kumar (2008)
		Indirect: *M. polymorpha* metabolites against bacteria *in vitro*	*M. polymorpha* metabolites (such as marchantin A) have antibacterial capacity against gram-positive bacteria.	Ivković et al. (2021)
	*Streptococcus pyrogenes*	Indirect: *M. polymorpha* metabolites against bacteria *in vitro*	*M. polymorpha* metabolites (such as marchantin A) have antibacterial capacity	Kámory et al. (1995)
	*Xanthomonas oryzae* pv. *oryzae*	Indirect: *M. polymorpha* metabolites against bacteria *in vitro*	*M. polymorpha* methanol and chloroform extracts have antibacterial capacity	Gahtori and Chaturvedi (2011)
	Many different species	Direct contact	Characterization of endophytic bacteria diversity	Alcaraz et al. (2018)
Fungi	*Alternaria solani*	Indirect: *M. polymorpha* metabolites against fungi *in vitro*	*M. polymorpha* methanol and flavonoid extracts have antifungal capacity	Mewari and Kumar (2011)
	*Aspergillus fumigatus*	Indirect: *M. polymorpha* metabolites against fungi *in vitro*	*M. polymorpha* dimethyl sulfoxide extracts have antifungal capacity	Sabovljević et al. (2011)
	*Aspergillus versicolor*	Indirect: *M. polymorpha* metabolites against fungi *in vitro*	*M. polymorpha* dimethyl sulfoxide extracts have antifungal capacity	Sabovljević et al. (2011)
	*Bicogniauxia mediterranea*	Direct contact	Isolation of endophytic fungi and characterization of their plant growth promoting ability	Nelson et al. (2018)
	*Bjerkandera adusta*	Direct contact	Isolation of pathogenic fungi and characterization of defensive response	Matsui et al. (2020)
	*Candida albicans*	Indirect: *M. polymorpha* metabolites against fungi *in vitro*	*M. polymorpha* methanol extracts have antifungal capacity	Mewari and Kumar (2008)
	*Colletotrichum truncatum*	Direct contact	Isolation of endophytic fungi and characterization of their plant growth promoting ability	Nelson et al. (2018)
	*Daldinia loculata*	Direct contact	Isolation of endophytic fungi and characterization of their plant growth promoting ability	Nelson et al. (2018)
	*Fusarium oxysporum*	Indirect: *M. polymorpha* metabolites against fungi *in vitro*	*M. polymorpha* methanol and flavonoid extracts have antifungal capacity	Mewari and Kumar (2011)
	*F. oxysporum* f. sp. *lini*	Indirect: *M. polymorpha* metabolites against fungi *in vitro*	*M. polymorpha* methanol and chloroform extracts have antifungal capacity	Gahtori and Chaturvedi (2011)
	*F. oxysporum* f. sp. *lycopersici*	Direct contact	Early root colonization effectors are an evolutionarily conserved mechanism for multihost colonization by root infecting fungi	Redkar et al. (2022a)
		Direct contact	Characterization of conserved and dispensable fungal pathogenicity factors for multihost disease development by root infecting fungi	Redkar et al. (2022b)
	*Hypoxylon* sp.	Direct contact	Isolation of endophytic fungi and characterization of their plant growth promoting ability	Nelson et al. (2018)
	*Irpex lacteus*	Direct contact	Isolation of pathogenic fungi and characterization of defensive response	Matsui et al. (2020)
	*Microsphaeropsis arundinis*	Direct contact	Isolation of endophytic fungi and characterization of their plant growth promoting ability	Nelson et al. (2018)
	*Nemania* sp.	Direct contact	Isolation of endophytic fungi and characterization of their plant growth promoting ability	Nelson et al. (2018)
	*Nemania serpens*	Direct contact	Isolation of endophytic fungi and characterization of their plant growth promoting ability	Nelson et al. (2018)
	*Penicillium funiculosum*	Indirect: *M. polymorpha* metabolites against fungi *in vitro*	*M. polymorpha* dimethyl sulfoxide extracts have antifungal capacity	Sabovljević et al. (2011)
	*P. ochrochloron*	Indirect: *M. polymorpha* metabolites against fungi *in vitro*	*M. polymorpha* dimethyl sulfoxide extracts have antifungal capacity	Sabovljević et al. (2011)
	*Phaeophlebiopsis peniophoroides*	Direct contact	Isolation of pathogenic fungi and characterization of defensive response	Matsui et al. (2020)
	*Rhizoctonia solani*	Indirect: *M. polymorpha* metabolites against fungi *in vitro*	*M. polymorpha* dimethyl sulfoxide extracts have antifungal capacity	Sabovljević et al. (2011)
		Direct contact Indirect: cell free fungal filtrates and vollatiles	Description of a generalist plant pathogen as a nonpathogen of *M. polymorpha*	Poveda et al. (2023)
	*Rhizophagus fasciculatus*	Direct contact	Arbuscular mycorrhizal fungi behaved as a plant pathogen in the early evolutionary stages of terrestrial plants, and it was the development of a salicylic acid-mediated defensive response that allowed its mutualistic plant symbiosis	Poveda (2020b)
	*Sclerotinia delphinii*	Direct contact	First description as *M. polymorpha* pathogen	Tamura et al. (2019)
	*Sclerotium rolfsii*	Indirect: *M. polymorpha* metabolites against fungi *in vitro*	*M. polymorpha* methanol and chloroform extracts have antifungal capacity	Gahtori and Chaturvedi (2011)
	*Trichoderma* spp.	Direct contact Indirect: cell free fungal filtrates and volatiles	*Trichoderma* behaved as a plant pathogen in the early evolutionary stages of terrestrial plants, and it was the development of a salicylic acid-mediated defensive response that allowed its mutualistic plant symbiosis	Poveda et al. (2023)
	*Trichoderma* *viride*	Indirect: *M. polymorpha* metabolites against fungi *in vitro*	*M. polymorpha* dimethyl sulfoxide extracts have antifungal capacity	Sabovljević et al. (2011)
	*Trychophyton mentagrophytes*	Indirect: *M. polymorpha* metabolites against fungi *in vitro*	*M. polymorpha* methanol extracts have antifungal capacity	Mewari and Kumar (2008)
	Many different species	Direct contact	Characterization of endophytic fungi diversity	Nelson and Shaw (2019)
Oomycete	*Phytophthora palmivora*	Direct contact	Characterization of the evolutionary infective mode of an oomycete	Carella et al. (2018)
–	Not identified	Direct contact	Characterization of anticancer compounds from endophytic microorganisms of *M. polymorpha.*	Stelmasiewicz et al. (2022)
		Direct contact	Characterization of anticancer and antiviral compounds from endophytic microorganisms of *M. polymorpha.*	Stelmasiewicz et al. (2023b)

### 5.1 Antimicrobial metabolites produced by *M. polymorpha*

One of the extensively studied aspects of *M. polymorpha*–microorganism interactions is their potential exploitation as a source of antimicrobial secondary metabolites. Numerous studies have demonstrated the ability of liverwort to produce and accumulate antiviral, antibacterial, and antifungal metabolites in its tissues, exhibiting activity against plant and animal pathogens. In 1998, Frame et al. obtained ethanol extracts from the leaves of different plant species in Puerto Rico, revealing that extracts obtained from *M. polymorpha* inhibited the growth of *Mycobacterium smegmatis*. This suggests that *M. polymorpha* could be a valuable resource for obtaining effective anti-*Mycobacterium tuberculosis* substances, a globally distributed pathogen with a high capacity to develop resistance to antibiotics (Frame et al., 1998). However, the metabolites extracted from M*. polymorpha* are not effective against all pathogenic bacteria. For instance, methanolic extracts obtained from *M. polymorpha* had inhibitory effects against the non-pathogenic bacterium *Bacillus subtilis* but were ineffective against the animal pathogenic bacteria *Staphylococcus aureus*, *Escherichia coli*, *Enterococcus faecalis*, and *Pseudomonas aeruginosa*, as well as the pathogenic yeast *Candida albicans* (Yayintas et al., 2019). The efficacy of the extracts might be influenced by the extraction method rather than the presence or absence of antimicrobial compounds in *M. polymorpha* tissues. This is the case of the work done by Mewari and Kumar (2008), where methanol and flavonoid extracts were reported to be potent antibacterial against *E. coli*, *S. aureus*, and *Proteus mirabilis*, and antifungal against *C. albicans* and the filamentous fungal skin pathogen *Trychophyton mentagrophytes*. However, they are ineffective against other filamentous fungal pathogens, such as *Aspergillus flavus* and *A. niger* (Mewari and Kumar, 2008).

Several studies have explored the combination of *M. polymorpha* extracts against animal and plant pathogens. Methanol and chloroform extracts from *M. polymorpha* have been identified as potent antibacterials against the animal pathogen *Pasteurella multocida* and the plant pathogen *Xanthomonas oryzae* pv. *oryzae*, as well as potent antifungal agents against the plant pathogens *F. oxysporum* f. sp. *lini* and *Sclerotium rolfsii*. However, these extracts were non-effective against the animal pathogenic bacterium *Salmonella enterica* and the plant pathogenic fungus *Tilletia indica* (Gahtori and Chaturvedi, 2011). Notably, dimethyl sulfoxide extracts from *M. polymorpha* exhibit unique antifungal activity against filamentous fungi. They proved effective against respiratory pathogens, such as *Aspergillus versicolor* and *Aspergillus fumigatus*, the postharvest fruit pathogen *Penicillium funiculosum*, and even beneficial fungi used as biological control agents, such as *Penicillium ochlorochloron* or *Trichoderma viride* (Sabovljević et al., 2011). However, it is essential to highlight that only one study has conducted both *in vitro* and *in vivo* assessments, representing a notable limitation. In the study developed by Mewari and Kumar (2011), they described, in the first stage, the antifungal capacity of methanol and flavonoid extracts from *M. polymorpha* against plant pathogens, inhibiting *Rhizoctonia solani* micellar growth, and the germination of spores of *Alternaria alternata* and *F. oxysporum*. In addition, these extracts were applied to eggplant (*Solanum melongena*) seeds, increasing seed germination and vigor, thereby decreasing the percentage of pathogen infection (Mewari and Kumar, 2011).

Although several studies have explored the antimicrobial capabilities of *M. polymorpha* metabolites, only a few have successfully identified these chemical compounds. This is the case for the metabolite marchantin A, a cyclic bis(bibenzyl ether) (a phenylpropanoid) widely accumulated in liverworts but more markedly in *M. polymorpha* (PubChem, 2023). This metabolite has been described as a potent antibacterial agent against pathogenic bacteria in animals and humans. However, the mode of action and specificity of this compound remain unclear. The results obtained with marchantin A are very different in the two currently available works existing up to now. In 1995, Kámory et al., described the antibacterial capacity of marchantin A against some gram-negative animal pathogenic bacteria (*Haemophylus influenzae*, *Neisseria meningitidis*, *P. multocida*, and *P. aeruginosa*) and gram-positive bacteria (*S. aureus* and *Streptococcus pyrogenes*). However, it was not effective against gram-negative *E. coli*, Gram-positive *Streptococcus viridans*, and S*. faecalis* (Kámory et al., 1995). In a more recent 2021 study by Ivković et al., marchantin A was found to be effective only as an antibacterial agent against harmless gram-positive bacteria, such as *B. subtilis*, animal pathogens, such as *Listeria monocytogenes* and *S. aureus*, and plant pathogens, such as *Clavibacter michiganensis*, but not against gram-negative bacteria, such as *P. aeruginosa*, *E. coli*, *P. syringae*, and *Xanthomonas arboricola* (Ivković et al., 2021). These disparities highlight the need for further research to understand the mode of action and efficacy of this metabolite. Another metabolite identified in *M. polymorpha* extracts is pheophorbide A, derived from chlorophyll, and has been described as a potent antiviral compound. This metabolite is a porphyrin compound that was studied during the COVID-19 health crisis, against its causative virus, severe acute respiratory syndrome coronavirus 2 (SARSCoV-2). The study reported that pheophorbide A interferes with the viral particle, preventing infection of cultured monkey and human cells by SARS-CoV-2 without noticeable cytotoxicity. Furthermore, the metabolite demonstrated efficacy against other RNA viruses, including the hepatitis C virus, West Nile virus, and other coronaviruses (Jimenez-Aleman et al., 2021).

### 5.2 Identification and characterization of epiphytic, endophytic, and pathogenic microorganisms from *M. polymorpha*

In recent years, numerous studies have focused on the isolation and characterization of microorganisms associated with *M. polymorpha*, a previously under-studied field. These investigations have led to the description of new microbial species and new biological functions for the previously described species. In this sense, the use of current massive sequencing methodologies has allowed the description of *M. polymorpha* endophytic microbiota. In different samples of this liverwort growing wild, a group of bacterial genera *Methylobacterium*, *Rhizobium*, *Paenibacillus*, *Lysobacter*, *Pirellula*, *Steroidobacter*, and *Bryobacter* present in all samples were identified. These bacterial genera play crucial biological functions in their host plants, such as plant growth promotion, complex exudate degradation, nitrogen fixation, methylotrophs, and disease-suppressive bacteria (Alcaraz et al., 2018). Similarly, another study identified the core mycobiome associated with *M. polymorpha* in different populations distributed throughout the United States. These endophytic fungi with possible important biological roles in *M. polymorpha* are *Candida sake*, *Hypoxylon submonticulosum*, *Nemania* sp., *Phoma herbarum*, and *Xylaria cubensis* (Nelson and Shaw, 2019). The isolation and cultivation of these endophytic fungi have provided insights into their potential biological effects when reintroduced into their host plants. For instance, the fungal species *Bicogniauxia mediterranea*, *Colletotrichum truncatum*, *Daldinia loculata*, *Hypoxylon* sp., *Microsphaeropsis arundinis*, and *Nemania serpens* have been described as plant growth promoters for *M. polymorpha*. In contrast, *Phoma herbarum*, *Toxicocladosporium irritans*, and *Colletotrichum acutatum* species showed no significant effect on the growth and development of their host plants, while *Hypoxylon submonticulosum* and *X. cubensis* species exhibited pathogenic effects (Nelson et al., 2018).

However, experimental studies have focused on the isolation of endophytic microorganisms from *M. polymorpha* with potential applications in different industries. These microorganisms have demonstrated the ability to produce antiviral and anticancer compounds, such as the volatile diketopiperazine derivatives, cyclo(L-phenylalanyl-L-prolyl) and cyclo(L-leucyl-L-prolyl). Both compounds have been described as effective anviral agents against herpesvirus type-1 and anticarcinogens in hypopharyngeal squamous cell carcinoma and cervical adenocarcinoma (Stelmasiewicz et al., 2022; Stelmasiewicz et al., 2023b).

In relation to epiphytic microorganisms, a novel pink-pigmented and facultative methylotrophic bacterial species, *Methylobacterium marchantiae* sp. nov., has been isolated and described (Schauer et al., 2011). This bacterium can stimulate the surface expansion of gemmae isolated from *M. polymorpha* by about 350% and form dense bacteria clusters that allow them to survive during periods of drought (Kutschera et al., 2007).

Isolation of pathogenic microorganisms from *M. polymorpha* tissues provides a deeper understanding of how this plant defends itself against its enemies. In a recently published article, three pathogenic fungal species of *M. polymorpha* have been described: *I. lacteus*, *Phaeophlebiopsis peniophoroides*, and *Bjerkandera adusta*. Subsequently, the use of *I. lacteus* in combination with exogenous application of salicylic acid (SA) or bioactive jasmonate in *M. polymorpha*, dinor-cis-12-oxo-phytodienoic acid (dn-OPDA), provided insights into the hormonal pathway involved in the activation of plant defenses against this pathogen. The study identified that the oxylipin pathway, which is antagonistic to the SA pathway, plays a key role in triggering plant defense against *I. lacteus* (Matsui et al., 2020). Oxylipins are plant molecules well documented for their role in plant defenses against pathogen attack, both directly (by damaging microbial cell membranes) and as signaling molecules in the activation of systemic plant resistance (Deboever et al., 2020).

### 5.3 Molecular studies of the direct interaction between *M. polymorpha* and microorganisms

The field of study focusing on the molecular interaction between *M. polymorpha* and microorganisms has seen significant development in the last 5 years; however, it is important to note that all these studies have been carried out with microorganisms not isolated from the liverwort tissues. The initial study involved the broad host-range oomycete pathogen *P. palmivora*, shedding light on the ancient plant trait of the intracellular accommodation of filamentous microbes. In the *M. polymorpha*–*P. palmivora* interaction, the pathogen establishes a complex tissue-specific interaction with the plant, completing its life cycle within the air chamber of the dorsal photosynthetic layer. In addition, *P. palmivora* invaginates *M. polymorpha* cells with haustoria-like structures that accumulate the cellular transport and membrane synthesis machinery of the host plant. This study contributes to our understanding of the interactions between filamentous pathogens and early divergent land plants (Carella et al., 2018).

Experimental investigations have delved into understanding the evolutionary dynamics of plant–microorganism interactions, termed evolutionary molecular plant–microbe interactions (EvoMPMI). Studies have been conducted on pathogenic bacteria, focusing on the hemi-biotrophic pathogenic bacterium *P. syringae* pv. tomato. This bacterium is capable of colonizing *M. polymorpha* tissues (Matsumoto et al., 2022) and triggers an effective defensive response against the pathogen (Gimenez-Ibanez et al., 2019). This defensive response is based on hormonal signaling carried out by SA, a signaling pathway present in this plant group but absent in algae. Notably, hormonal signaling is entirely conserved from the earliest terrestrial colonizing plants to the most complex angiosperms (Gimenez-Ibanez et al., 2019).

Specifically, SA-mediated hormonal signaling is considered the necessary basis for pathogenic fungi of *M. polymorpha* to behave as beneficial fungi in higher plants. In a study published in 2020, the pathogenic behavior of arbuscular mycorrhizal fungi (AMF), such as *Rhizophagus fasciculatus* was demonstrated against *M. polymorpha*. These fungi colonize tissues, inhibit their growth, and cause clear signs of disease, including decreased vitality and increased accumulation of reactive oxygen species. Although AMF are widely recognized for their role as agricultural bioinoculants, providing nutrients and water to host plants, the study reported no nutrient contribution by *R. fasciculatus* in colonizing *M. polymorpha* tissues. Furthermore, the study highlights that in the presence of SA signaling, AMFs are transformed into beneficial microorganisms for the plant (Poveda, 2020b). In relation to the AMF–*Marchantia* interaction, it is important to highlight that *M. polymorpha* lacks some of the genes for mycorrhizal symbiosis, being a non-AMF host, unlike other species of the genus, such as *M. paleacea* (Vigneron et al., 2018; Genre et al., 2020).

Building on the same premise, another study investigated the interaction between a beneficial endophytic filamentous fungus, such as the genus *Trichoderma*, and *M. polymorpha*, as well as with other plants representing subsequent steps in plant evolution, such as the pteridophyte *Dryopteris affinis* and the angiosperm *Arabidopsis thaliana*. By studying the direct fungus–plant interaction and the indirect interaction through diffusible and/or volatile secondary metabolites, the species *Trichoderma virens*, *T. brevicompactum* and *T. hamatum* were described as pathogens by colonization of *M. polymorpha* tissues, and the species *T. asperellum* as a producer of toxic metabolites harmful for the liverwort. However, none of the *Trichoderma* species used in this study was pathogenic to pteridophytes or angiosperms. Subsequently, through transcriptomic analysis and exogenous application of SA, we identified how this hormone induces *M. polymorpha* defenses against the pathogenic species of *Trichoderma*. Consequently, these fungi begin to behave as endophytes. These findings suggest that *Trichoderma* may have undergone an evolutionary period of interaction with plants, in which it initially acted as a plant pathogen until plants developed a defense system, mediated by SA, to restrict its colonization (Poveda et al., 2023).

Other studies have focused on the molecular studies of the pathogen and its role in *M. polymorpha* infection. The tomato root fungus *F. oxysporum* f. sp. *lycopersici* can cause tissue maceration and host cell death in *M. polymorpha*. For this fungus to act as a liverwort pathogen, the action of early root colonization effectors and other pathogenicity factors, such as mitogen-activated protein kinases, transcriptional regulators, and cell wall remodeling enzymes is crucial. Furthermore, all these molecular mechanisms are necessary for pathogenic fungi to infect the roots of higher plants and are conserved in other species of root pathogens. Therefore, the molecular mechanisms required for the infection of *M. polymorpha* by *F. oxysporum* are evolutionarily conserved for multihost colonization by root-infecting fungi (Redkar et al., 2022a; Redkar et al., 2022b).

### 5.4 *M. polymorpha* transformation by means of bacterial vectors

The most widely adopted methodology for both stable and transient transformation of plant species involves the use of *Agrobacterium tumefaciens* as a vector (Sutradhar and Mandal, 2023). Numerous studies have been conducted on the transformation of *M. polymorpha* through its interaction with *A. tumefaciens*. Pioneering work was published in 1997, where stable transformation of *M. polymorpha* with the *GUS* (*β-glucuronidase*) gene was achieved (Nasu et al., 1997). Subsequently, in addition to a reporter (such as *GUS*), transformation was achieved with a selection marker, the *hygromycin phosphotransferase* (*HPT*) gene (Ishizaki et al., 2008), as well as *gentamicin 3’-acetyltransferase* gene (Ishizaki et al., 2015). Contemporary gene editing techniques, such as CRISPR/Cas9, have also been tested using *A. tumefaciens* as a vector (Sugano et al., 2014). The transient transformation of *M. polymorpha* tissues has also been achieved using this bacterial vector. This is the case for 2–3 days of transient expression of the GUS gene through *M. polymorpha*-*A. tumefaciens* coculture (Iwakawa et al., 2021).

When comparing the efficiency of stable transformation of *M. polymorpha* using the bacterium *A. tumefaciens* as a vector with other model plant species, the results were notably positive without tissue damage. The direct transformation of sporangium spores in *M. polymorpha* has been reported to be 1.75% (Ishizaki et al., 2008). This efficiency, achieved without causing tissue damage, was similar to the reported efficiency of *A. tumefaciens*-mediated floral dip transformation in *A. thaliana* (± 1%) (Zhang et al., 2006). However, both efficiencies were significantly lower than those attainable through tissue transformation with *A. tumefaciens*, followed by regeneration of the entire plant. In *A. thaliana*, this method achieves efficiencies ranging from 80% to 90% (Akama et al., 1992). However, this technique has its own set of disadvantages, including higher labor requirements and the potential for somaclonal variation.

## 6 Conclusions and future perspectives

Significant advancements in the understanding of *M. polymorpha*–microorganism interactions have occurred in recent years, spanning both evolutionary and applied perspectives. Notably, the most extensively explored facet within this domain is the synthesis of antimicrobial compounds using *M. polymorpha*. It has been established as a prolific source of effective compounds for combatting crucial plant and animal pathogens, including the formidable human tuberculosis bacterium (*M. tuberculosis*). However, the effectiveness of extracts derived from *M. polymorpha* varies, necessitating further investigation to identify the specific compounds involved and the optimal culture conditions that maximize their production and accumulation within plant tissues. Furthermore, most studies conducted thus far are *in vitro*, underscoring the imperative for the development of *in vivo* studies involving actual pathogenic infections to provide a more comprehensive understanding of the dynamics at play.

On the other hand, *M. polymorpha* serves as a valuable source for isolating not only new strains but also new species of epiphytic, endophytic, and pathogenic microorganisms. Moreover, these isolates exhibited biologically significant activities relevant to the agricultural and forestry sectors, such as atmospheric nitrogen fixation, production of plant growth factors, and antagonism against pathogens. In addition, they show promise in the pharmaceutical sector as producers of antiviral and anticancer compounds. Therefore, there is a need for future research focused on obtaining and characterizing new microbial isolates from *M. polymorpha*.

Molecular studies on the interaction of *M. polymorpha* with microorganisms, both endogenous and isolated from tissues of other plants, have provided valuable insights into the evolutionary dynamics of plant–microorganism interactions. These studies have shed light on various aspects, including the initiation of infection by oomycetes and pathogenic fungi in terrestrial plants, the antagonistic plant defensive response to SA by oxylipins against pathogens, and the importance and emergence of the plant defensive response to SA. Notably, in the absence of this response, beneficial microorganisms, such as mycorrhizal fungi or *Trichoderma*, can exhibit pathogenic behavior. This field of study is relatively new, evolving, and promising to contribute substantial knowledge in the years to come.

Given its significance and widespread use as a model plant, numerous studies have been conducted to establish precise and effective methodologies for plant transformation using *A. tumefaciens*, resulting in a standardized technique.

In summary, *M. polymorpha* has been widely used in recent years as a model plant in the interaction with microorganisms from a basic science point of view, such as the knowledge of the evolutionary plant–microorganism interaction, both beneficial and pathogenic. Furthermore, *M. polymorpha* is a plant widely used from a more practical point of view in its interaction with microorganisms, such as obtaining plant antimicrobial metabolites, isolation, and characterization of new microorganisms, or their use in genetic engineering.

## Author contributions

JP: Conceptualization, Investigation, Methodology, Supervision, Writing – original draft, Writing – review & editing.
